# Meta-analysis of the diagnostic value of exosomal microRNAs in renal cell carcinoma

**DOI:** 10.3389/fonc.2024.1441429

**Published:** 2024-10-28

**Authors:** Qingru Li, Jing Tian, Cuiqing Chen, Hong Liu, Binyi Li

**Affiliations:** ^1^ Department of Nephrology, the Eighth Clinical Medical School of Guangzhou University of Chinese Medicine, Foshan, China; ^2^ Department of Nephrology, Foshan Hospital of Traditional Chinese Medicine, Foshan, China; ^3^ Department of Cardiovascular, the First Clinical Medical College of Henan University of Chinese Medicine, Zhengzhou, China; ^4^ Department of Oncology, Shenzhen Bao’an Authentic TCM Therapy Hospital, Shenzhen, China

**Keywords:** exosomal microRNAs, renal cell carcinoma, miRNA, meta-analysis, diagnosis

## Abstract

**Aim:**

This meta-analysis aims to evaluate the potential of exosomal microRNAs(Exo-miRs) as diagnostic biomarkers for renal cell carcinoma(RCC).

**Methods:**

Clinical studies reporting the use of Exo-miRs in the diagnosis of RCC were retrieved from PubMed, Web of Science, Cochrane Library, Embase, China National Knowledge Infrastructure (CNKI), Wanfang, VIP, and Chinese Biomedical Literature Database (SinoMed). After relevant data were screened and extracted, the quality of the included studies was assessed using the QUADAS-2 tool. The Meta-disc (version 1.4) software was used to analyze the heterogeneity of threshold/non-threshold effects in the included studies. The Stata MP (version 16.0) software was used to calculate sensitivity(Sen), specificity(Spe), positive likelihood ratio(+LR), negative likelihood ratio(-LR), area under the curve(AUC), diagnostic odds ratio(DOR), and publication bias.

**Results:**

A total of 11 studies were included in this meta-analysis. Spearman correlation coefficient was 0.319 (*P* = 0.075; >0.05), indicating no threshold effects. The pooled Sen, Spe, +LR, -LR, DOR, and AUC were 0.73 (95% *CI*, 0.68–0.78), 0.81 (95% *CI*, 0.76–0.85), 3.80 (95% *CI*, 3.02–4.77), 0.33 (95% *CI*, 0.28–0.40), 11.48 (95% *CI*, 8.27–15.95), and 0.84 (95% *CI*, 0.80–0.87), respectively. No publication bias was detected among the included studies.

**Conclusion:**

The expression of Exo-miRs plays an important role in the diagnosis of RCC. However, owing to the limited number of included studies and heterogeneity among them, further clinical research is necessary to verify the findings of this meta-analysis.

**Systematic review registration:**

https://www.crd.york.ac.uk/PROSPERO, identifier CRD42023445956.

## 1 Introduction

Renal cell carcinoma (RCC), commonly known as kidney cancer, originates from renal tubular epithelial cells. It is a common malignant tumor of the urinary system, accounting for 80%–90% of all malignant tumors originating from the kidney. The incidence of RCC is increasing worldwide, with cases accounting 2%–3% approximately of all adult malignancies (1), and varies across regions, with the highest incidence rates being observed in developed western countries such as North America and Western Europe. Over the past 20 years, the latest Global Cancer Watch data show an annual 2% increase in RCC incidence with more than 400 000 new diagnoses and almost 180 000 deaths per year worldwide by 2020 (2). The kidney can compensate for impaired function to some extent, thus makes early kidney dysfunction difficult to detect. Approximately one-third of patients with RCC have metastatic lesions at diagnosis, whereas another one-third of patients may develop metastasis during the course of the disease (3). RCC is often asymptomatic and cannot be palpated in the early stages. It is diagnosed incidentally during imaging in >50% of cases (4). Ultrasonography is a common method for diagnosing kidney diseases. However, owing to the influence of bowel gas and tumor depth, its accuracy in diagnosing RCC is relatively low. Contrast-enhanced computed tomography (CECT) helps overcome the limitations but has the disadvantage of radiation exposure and other adverse events, such as allergic reactions and acute kidney injury (5). Blood-based biomarkers such as carcinoembryonic antigen and M2 pyruvate kinase have been evaluated for their potential in early detection of RCC. However, the low specificity and sensitivity limit their clinical utility. Early diagnosis and prognosis of advanced disease are key to improving the overall survival of patients with RCC. Therefore, non-invasive diagnostic methods with high sensitivity and specificity are urgently required to achieve early diagnosis and optimize disease management.

In recent years, owing to the advancement of biomedical technology, exosomes have received increased attention as potential biomarkers in RCC. Exosomes are membrane-bound vesicles formed through the fusion of multivesicular bodies with the cell membrane (6). They can carry various bioactive components, such as lipids, proteins, nucleic acids, and cellular metabolites, and have been found in various body fluids, such as cerebrospinal fluid, blood, urine, saliva, and breast milk (7). They play an important role in mediating intercellular communication through endocrine or paracrine mechanisms and hence act on target cells. miRNAs are the most abundant RNA class in exosomes. They can regulate gene expression at the post-transcriptional level by interacting with mRNAs (8). To date, more than 1400 human miRNAs have been identified, which are involved in the regulation of more than one-third of genes (9). Studies have shown that miRNAs are closely associated with the diagnosis, treatment, and prognosis of tumors (10). Owing to their resistance to endogenous ribonucleases, miRNAs are highly stable in various body fluids. Additionally, the ability of exosomes to maintain the stability of miRNAs allows them to exist more stably in the extracellular environment.

A large number of studies have shown that exosomal microRNAs(Exo-miRs) may have great potential in the diagnosis of RCC (11, 12). Exo-miRs not only have a close relationship with urogenital system tumors but also can reflect the miRNA expression profile of source cells. Therefore, they hold substantial promise in early detection, auxiliary staging, treatment response assessment, and prognosis prediction in urological tumors (13). However, studies on the abnormal expression and functional relevance of Exo-miRs in RCC are limited. Moreover, these studies are primarily conducted in different research institutions and hence lack consistent evidence-based results. Therefore, this meta-analysis aims to assess the diagnostic value of Exo-miRs in RCC, providing a more comprehensive reference for its early diagnosis.

## 2 Materials and methods

This meta-analysis followed the guidelines outlined in the PRISMA statement (14), and the detailed protocol has been registered in PROSPERO (https://www.crd.york.ac.uk/PROSPERO) (Registration number: CRD42023445956).

### 2.1 Search strategy

Two researchers independently searched for relevant articles in four English electronic databases, namely, PubMed, Embase, Cochrane Library, and Web of Science, and four Chinese electronic databases, namely, WanFang, VIP, China National Knowledge Infrastructure (CNKI), and Chinese Biomedical Literature Database (SinoMed). All relevant articles published before April 18, 2024, in English or Chinese were collected. The search terms included “exosomes”, “extracellular vesicles”, “kidney cancer”, “renal cell cancer”, and “renal cell carcinoma”. Subject term expansion was used during retrieval.

### 2.2 Inclusion criteria

The inclusion criteria were as follows: (1) Articles in which the experimental group comprised individuals with RCC and the control group comprised individuals without RCC (including individuals with kidney diseases other than RCC and healthy individuals); (2) Articles in which postoperative histopathological examination served as the gold-standard method for diagnosis; (3) Articles that involved the use of extracellular vesicle-derived miRNAs as diagnostic markers for RCC and provided data on sample size and true-positive (TP), false-positive (FP), false-negative (FN), and true-negative (TN) rates; and (4) articles for which the full text was available in either Chinese or English.

### 2.3 Exclusion criteria

The exclusion criteria were as follows: (1) Repetitively published articles; (2) comments, reviews, case studies, conference abstracts, and fundamental research; (3) articles without full-text availability or with missing data; (4) articles with a high risk of bias or a controversial design; and (5) articles with an unreasonable experimental design.

### 2.4 Data extraction

Based on the inclusion and exclusion criteria, two reviewers (L.Q. and T.J.) independently screened all articles to extract relevant data, including the name of the first author, publication year, country, sample size, histological results, detection methods, source of exosomes, Sen, Spe, and area under the curve(AUC) values. Outcome measures included the number of individuals with TP, FP, TN, and FN results. Disagreements were resolved through third-party adjudication (L.B. and L.H.).

### 2.5 Quality assessment

The methodological quality of the included articles was assessed using the Quality Assessment of Diagnostic Accuracy Studies (QUADAS-2) tool in the Review Manager (version 5.4) software (15). The QUADAS-2 tool consists of 14 items that are divided into two sections: risk of bias evaluation and clinical applicability. Each question is answered with “yes”, “no”, or “unclear”, and the risk of corresponding bias is determined as “low”, “high”, or “unclear”. If all signaling questions within a domain are answered with “yes”, the risk of bias is considered low. If any question is answered with “no”, the risk of bias is considered “high”.

### 2.6 Statistical analysis

The Meta-disc (version 1.4) software was used to assess threshold effects. If the Spearman correlation coefficient (*P*-value) between the logarithm of sensitivity and the logarithm of 1-specificity was >0.05, it indicated the absence of heterogeneity caused by a threshold effect. However, a *P*-value ≤ 0.05 suggested the presence of heterogeneity caused by a threshold effect (16). The MIDAS command in the Stata (version 16.0) software was used to fit a bivariate mixed-effect model and assess heterogeneity among the included articles. The level of heterogeneity was quantified using the *I^2^* value, in which an *I^2^* <50% indicated low heterogeneity and an *I^2^* ≥50% indicated high heterogeneity. If *I^2^* <50%, the fixed effect model is adopted. If *I^2^* ≥50%, a random effects model is used. In the case of high heterogeneity, subgroup analysis and meta-regression analysis were performed to determine the sources of heterogeneity. The evaluation indicators for combining diagnostic tests included Sen, Spe, +LR,-LR, AUC, and diagnostic odds ratio (DOR). Additionally, a Deeks’ funnel plot was generated to detect heterogeneity and publication bias among the included articles (17), and a Fagan’s nomogram was established to evaluate the role and clinical value of Exo-miRs in RCC. All statistical analyses were reviewed by biostatistics experts at Guangzhou University of Chinese Medicine.

## 3 Results

### 3.1 Literature search and study characteristics

**Figure 1:**
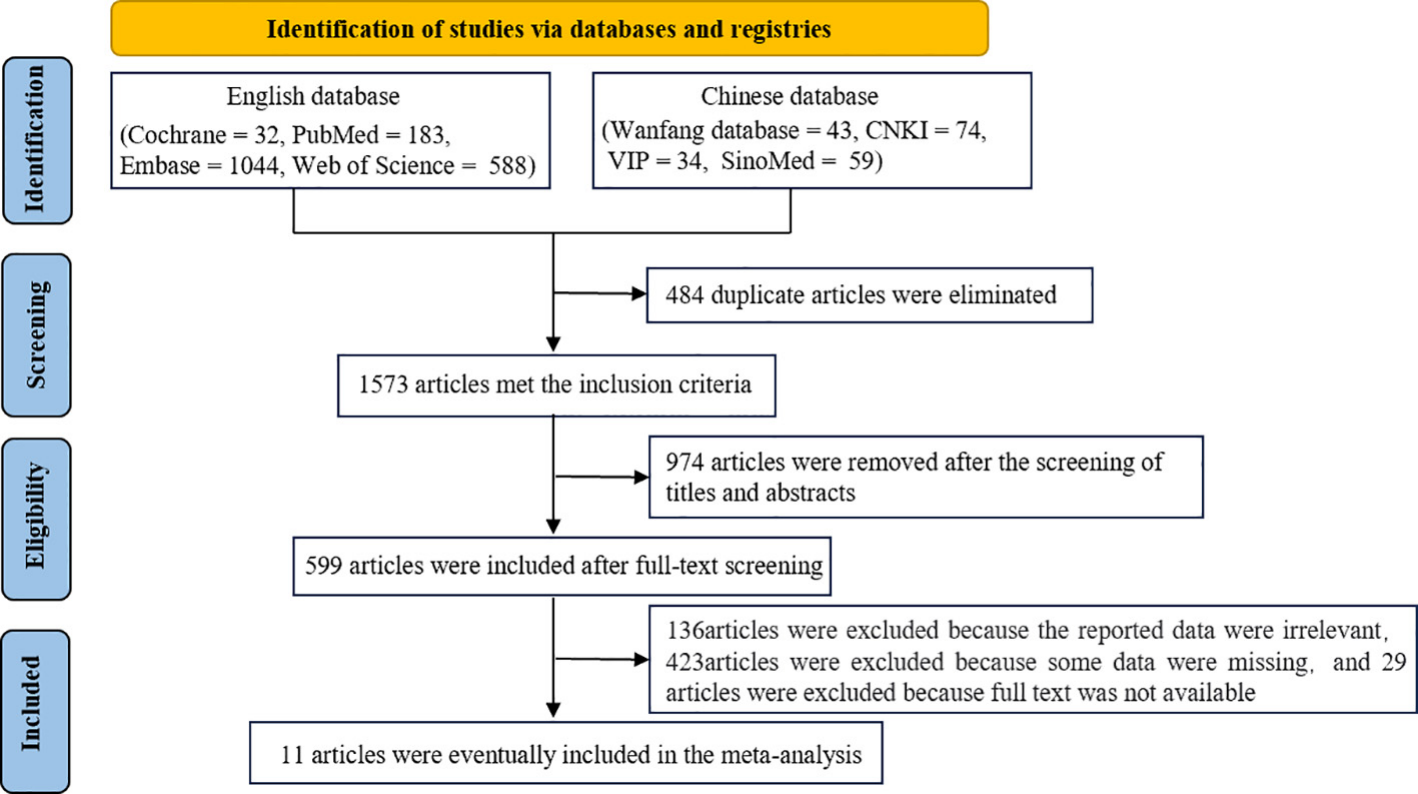
Flow diagram demonstrating the study selection process.

A total of 1573 articles were obtained after duplicate articles were eliminated. Of these articles, 1562 articles were excluded after screening. Specifically, 29 articles were excluded because the full text was not available owing to access rights and publisher restrictions. Eventually, 11 articles were included in this meta-analysis (18–28). The literature screening process is shown in **Figure 1**.

The characteristics of the included articles are shown in **Table 1**. Of the 11 included articles (18–28), 10 were journal articles (18–24, 26–28) and 1 was a master’s thesis (25). Six articles were published in Chinese (23–28) and five articles were published in English (18–22), with a total of 908 cases and 738 controls. Eight articles focused on clear cell renal cell carcinoma (ccRCC) (18, 20–22, 24, 26–28), whereas three articles focused on RCC (19, 23, 25). Three articles reported the use of urine-derived extracellular vesicles (18, 22, 25), one article reported the use of plasma-derived extracellular vesicles (19), and seven articles reported the use of serum-derived extracellular vesicles (20, 21, 23–28). In all included articles, the experimental group comprised patients with cancer confirmed via pathological examination, whereas the control group comprised individuals with benign renal lesions (renal cysts and hematomas), adjacent normal tissue samples, or healthy adults.

**Table 1:** Characteristics of included studies in this meta-analysis.

N0.	First author	Country	Language	Sample (n)		Source of control	Histology	Test method	Exosomal Source	miRNA Profile	Expression levels	AUC value	TP	FP	FN	TN	Sen	Spe
				Case Group	Control Group													
1	Shangqing Song2019 (18)	China	English	70	30	Healthy control	ccRCC	qRT-PCR	Urinary	miR-30c-5p	Down regulation	0.8192	48	0	22	30	68.57%	100%
2	Chu Tianxiao2020 (19)	China	English	22	16	Healthy individuals	RCC	qRT-PCR	Plasma	mir-149-3p	Up regulation	0.7188	17	4	5	12	75%	72.7%
3	Chu Tianxiao2020 (19)	China	English	22	16	Healthy individuals	RCC	qRT-PCR	Plasma	mir-424-3p	Up regulation	0.7727	19	3	3	13	75%	81.8%
4	Chu Tianxiao2020 (19)	China	English	22	16	Healthy individuals	RCC	qRT-PCR	Plasma	mir-92a-1-5p	Down regulation	0.8324	19	4	3	12	87.5%	77.3%
5	Xuegang Wang2018 (20)	China	English	45	30	Healthy volunteers	ccRCC	qRT‐PCR	Serum	miR-210	Up regulation	0.8779	37	6	8	24	82.5%	80%
6	Wei Zhang2016 (21)	China	English	82	80	Healthy volunteers	ccRCC	qRT‐PCR	Serum	miR-210	Up regulation	0.69	57	30	25	50	70%	62.2%
7	Wei Zhang2016 (21)	China	English	82	80	Healthy volunteers	ccRCC	qRT‐PCR	Serum	miR-1233	Up regulation	0.82	66	20	16	60	81%	76%
8	Henriett Butz2015 (22)	Canada	English	28	18	Healthy participants	ccRCC	qRT‐PCR	Urinary	miR-126-3p–miR-449a	Down regulation	0.84	23	7	5	11	83.8%	62.5%
9	Henriett Butz2015 (22)	Canada	English	28	18	Healthy participants	ccRCC	qRT‐PCR	Urinary	miR-126-3p–miR-34b-5p	Down regulation	0.79	22	5	6	13	77%	72.4%
10	Wang Cheng2018 (23)	China	Chinese	79	75	Healthy medical examiners	RCC	qRT‐PCR	Serum	miR-181a-3p	Up regulation	0.828	57	21	22	54	72.2%	72%
11	Sun Huaixin2023 (24)	China	Chinese	293	200	Healthy medical examiners	ccRCC	qRT‐PCR	Serum	miR-133a	Down regulation	0.78	150	0	143	200	53%	100%
12	Zhang Hongsen2022 (25)	China	Chinese	68	60	Patients with benign renal lesions	RCC	qRT‐PCR	Serum	miR-210	Up regulation	0.884	56	12	12	48	82.4%	80%
13	Zhang Hongsen2022 (25)	China	Chinese	68	60	Patients with benign renal lesions	RCC	qRT‐PCR	Serum	miR-21	Up regulation	0.738	37	11	31	49	54.4%	81.7%
14	Zhang Hongsen2022 (25)	China	Chinese	68	60	Patients with benign renal lesions	RCC	qRT‐PCR	Serum	miR-153	Up regulation	0.651	27	8	41	52	39.7%	86.7%
15	Zhang Hongsen2022 (25)	China	Chinese	68	60	Patients with benign renal lesions	RCC	qRT‐PCR	Serum	miR-1233	Up regulation	0.752	41	11	27	49	60.3%	81.7%
16	Zhang Hongsen2022 (25)	China	Chinese	68	60	Patients with benign renal lesions	RCC	qRT‐PCR	Serum	miR-221	Up regulation	0.928	54	3	14	57	79.4%	95%
17	Zhang Hongsen2022 (25)	China	Chinese	68	60	Patients with benign renal lesions	RCC	qRT‐PCR	Serum	miR-34a	Down regulation	0.865	60	19	8	41	88.2%	68.3%
18	Zhang Hongsen2022 (25)	China	Chinese	68	60	Patients with benign renal lesions	RCC	qRT‐PCR	Urinary	miR-210	Up regulation	0.868	57	17	11	43	83.8%	71.7%
19	Zhang Hongsen2022 (25)	China	Chinese	68	60	Patients with benign renal lesions	RCC	qRT‐PCR	Urinary	miR-21	Up regulation	0.915	55	7	13	53	80.9%	88.3%
20	Zhang Hongsen2022 (25)	China	Chinese	68	60	Patients with benign renal lesions	RCC	qRT‐PCR	Urinary	miR-153	Up regulation	0.626	37	20	31	40	54.4%	66.7%
21	Zhang Hongsen2022 (25)	China	Chinese	68	60	Patients with benign renal lesions	RCC	qRT‐PCR	Urinary	miR-1233	Up regulation	0.724	34	10	34	50	50%	83.3%
22	Zhang Hongsen2022 (25)	China	Chinese	68	60	Patients with benign renal lesions	RCC	qRT‐PCR	Urinary	miR-221	Up regulation	0.895	51	9	17	51	75%	85%
23	Zhang Hongsen2022 (25)	China	Chinese	68	60	Patients with benign renal lesions	RCC	qRT‐PCR	Urinary	miR-34a	Down regulation	0.961	58	7	10	53	85.3%	88.3%
24	Tian Yaping2018 (26)	China	Chinese	126	124	Healthy control	ccRCC	qRT‐PCR	Serum	miR-28-3p	Up regulation	0.800	85	30	41	94	67.7%	76.6%
25	Tian Yaping2018 (26)	China	Chinese	126	124	Healthy control	ccRCC	qRT‐PCR	Serum	miR-200a	Up regulation	0.780	99	48	27	86	78.5%	69.4%
26	Tian Yaping2018 (26)	China	Chinese	126	124	Healthy control	ccRCC	qRT‐PCR	Serum	miR-1826	Up regulation	0.817	110	49	16	75	87.1%	60.5%
27	Tian Yaping2018 (26)	China	Chinese	126	124	Healthy control	ccRCC	qRT‐PCR	Serum	miR-103	Up regulation	0.680	56	17	70	107	44.1%	86.3%
28	Tian Yaping2018 (26)	China	Chinese	126	124	Healthy control	ccRCC	qRT‐PCR	Serum	miR-1249	Up regulation	0.695	87	52	39	72	68.7%	58.1%
29	Tian Yaping2018 (26)	China	Chinese	126	124	Healthy control	ccRCC	qRT‐PCR	Serum	miR-640	Up regulation	0.662	57	13	69	101	45.2%	81.5%
30	Tian Yaping2018 (26)	China	Chinese	126	124	Healthy control	ccRCC	qRT‐PCR	Serum	miR-6-panel	Up regulation	0.832	106	21	20	103	83.9%	83.3%
31	Tian Yaping2021 (27)	China	Chinese	65	76	Healthy control	ccRCC	qRT‐PCR	Serum	miR-99b-3p	Up regulation	0.757	45	17	20	58	69.2%	77.3%
32	Wang Xuegang2017 (28)	China	Chinese	30	30	Healthy control	ccRCC	qRT‐PCR	Serum	miR-210	Up regulation	0.8789	28	6	2	24	92.1%	80%

RCC, renal cell carcinoma; ccRCC, clear cell renal cell carcinoma; TP, true positive; FP, false positive; FN, false negative; TN, true negative; Sen, Sensitivity; Spe, Specificity.

### 3.2 Risk of bias in included studies

**Figure 2:**
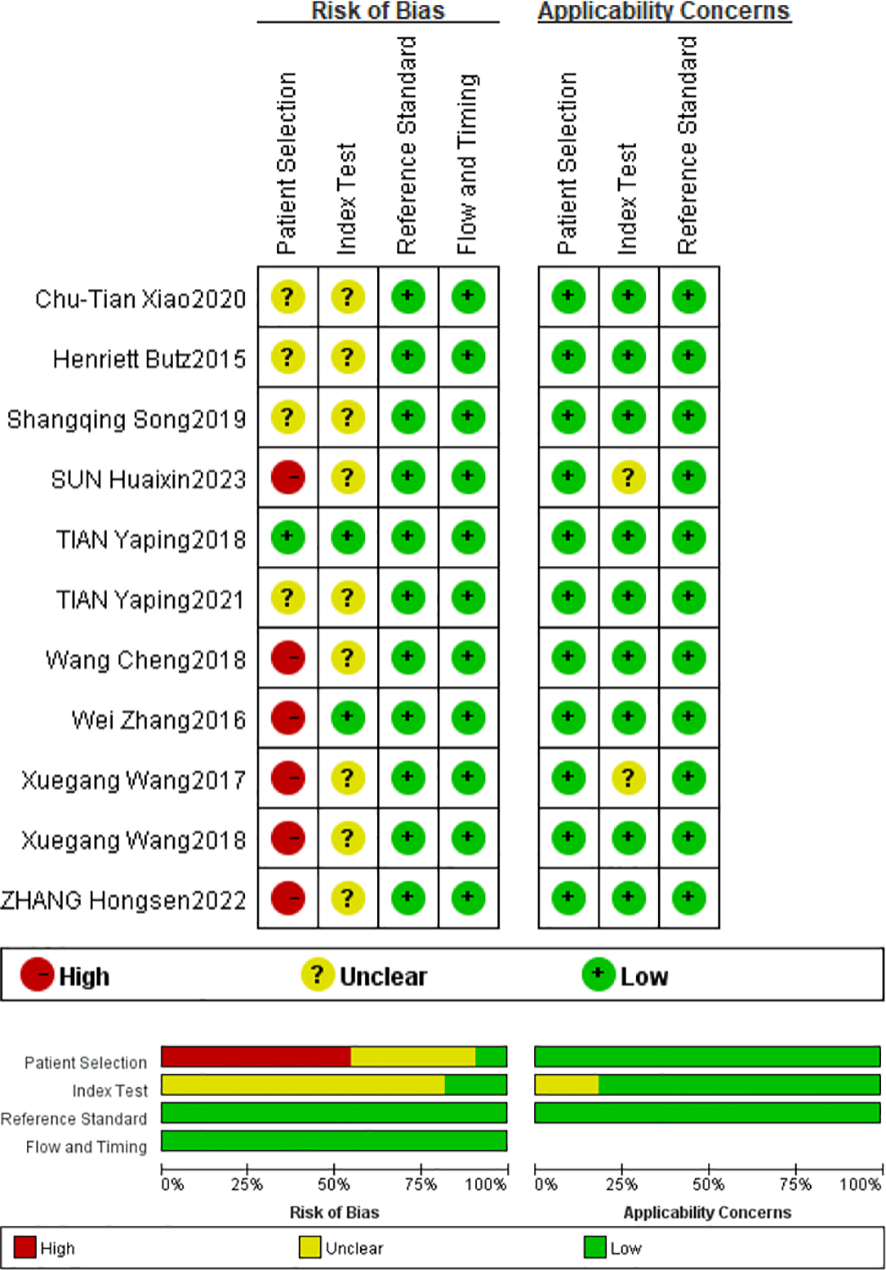
Detailed assessment of the risk of bias.

Based on QUADAS-2 scores, all included studies show a low risk of bias in the reference trial domain and the case process and progress domain, a high risk of bias in the patient selection domain, and an unclear risk of bias in the index domain. The risk of bias was primarily attributed to two aspects: the included articles were not randomized controlled trials and did not use prespecified thresholds. Overall, the methodological quality of the included articles was moderate to high, indicating that the articles were reliable. Based on QUADAS-2 scores, all included articles were identified to be of moderate to high quality (**Figure 2**).

### 3.3 Results of data analysis

The Spearman correlation coefficient was 0.319 (*P* = 0.075), indicating the absence of threshold effects. The *I^2^* values for pooled Sen and Spe were 89.14% and 85.65%, respectively, both *P* value were less than 0. 01, indicating the presence of heterogeneity. Therefore, the sources of heterogeneity were subsequently identified.

**Figure 3:**
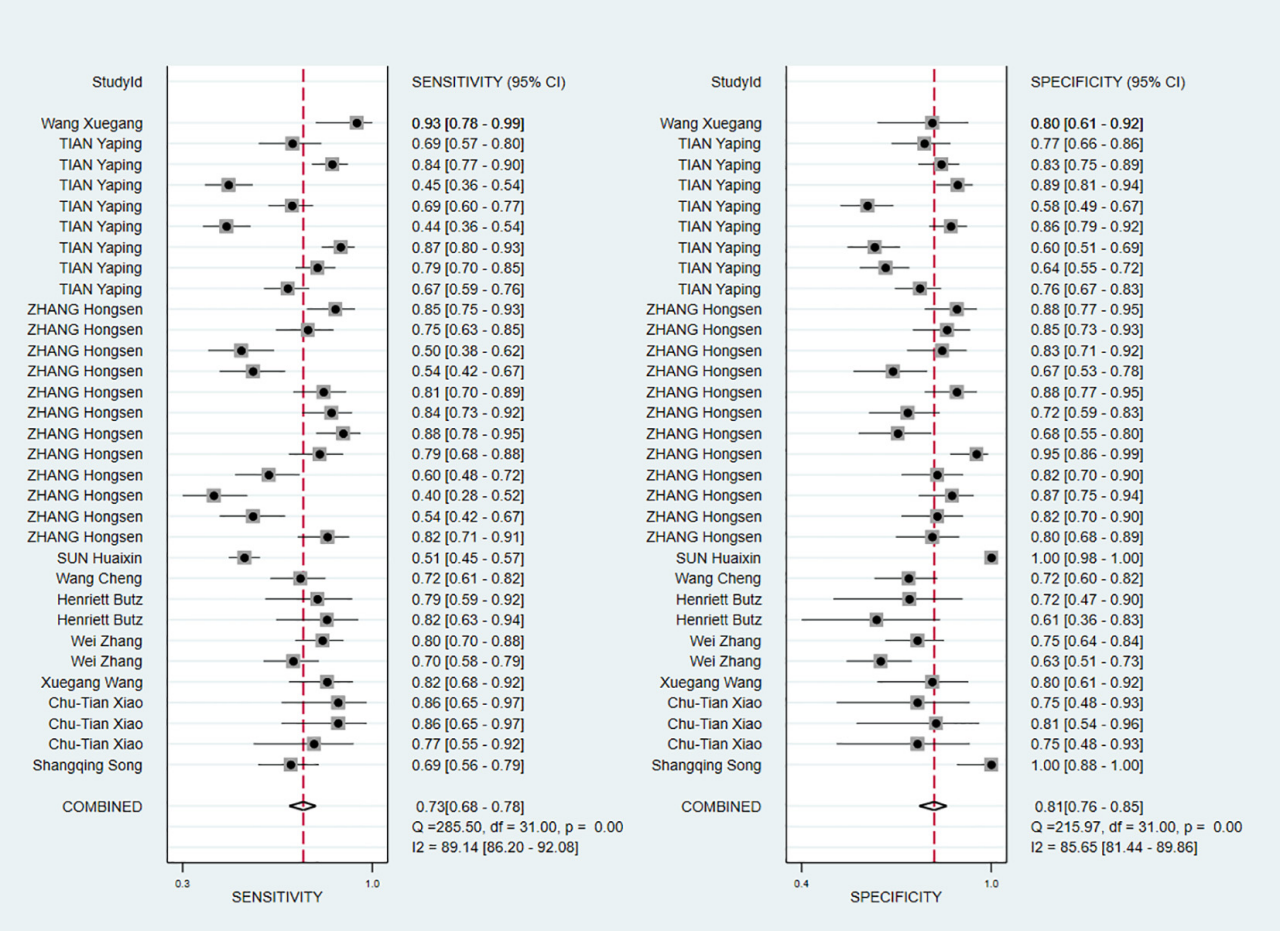
Forest plots demonstrating pooled Sen and Spe.

**Figure 4:**
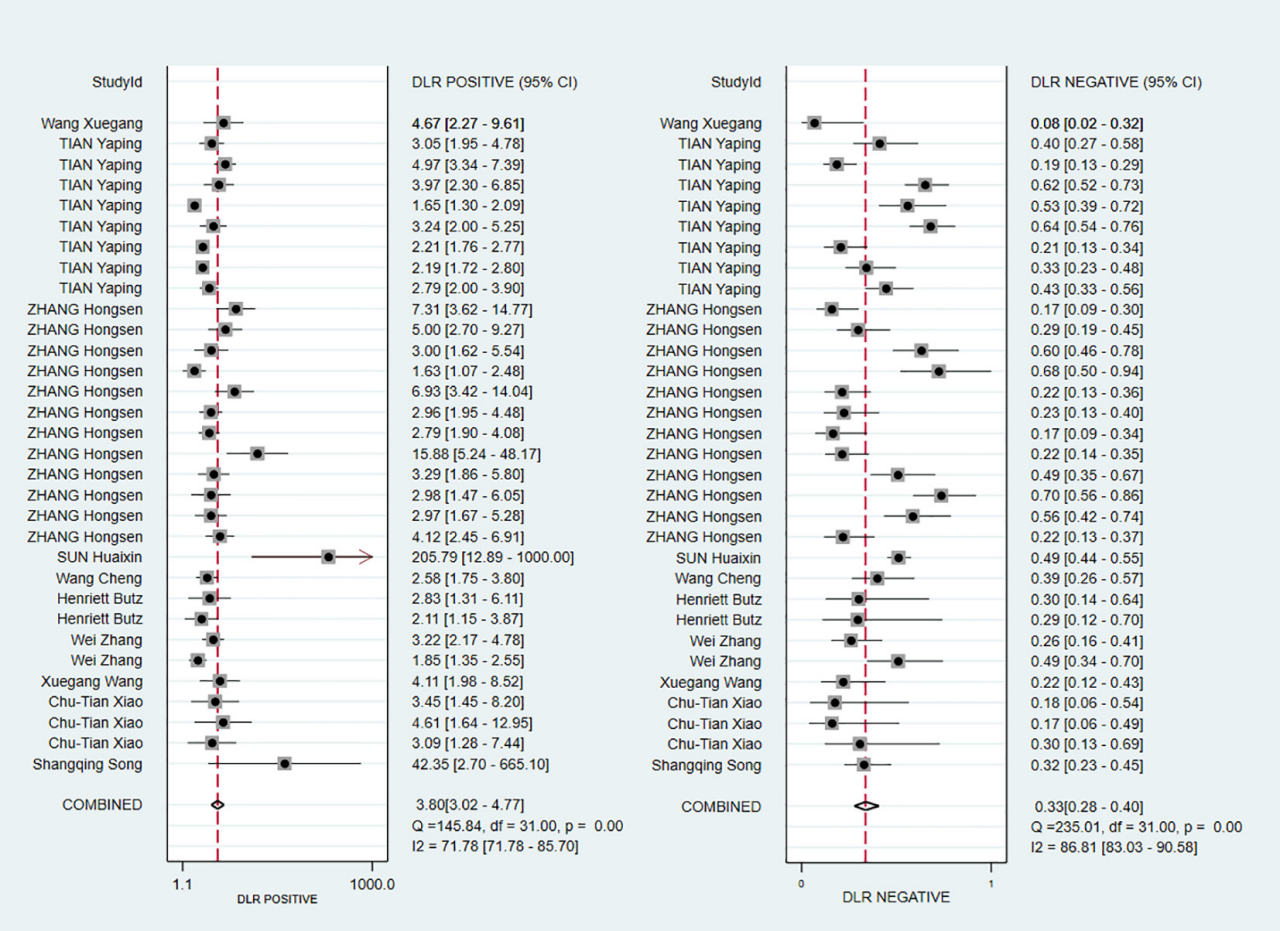
Forest plots demonstrating pooled +LR and –LR.

**Figure 5:**
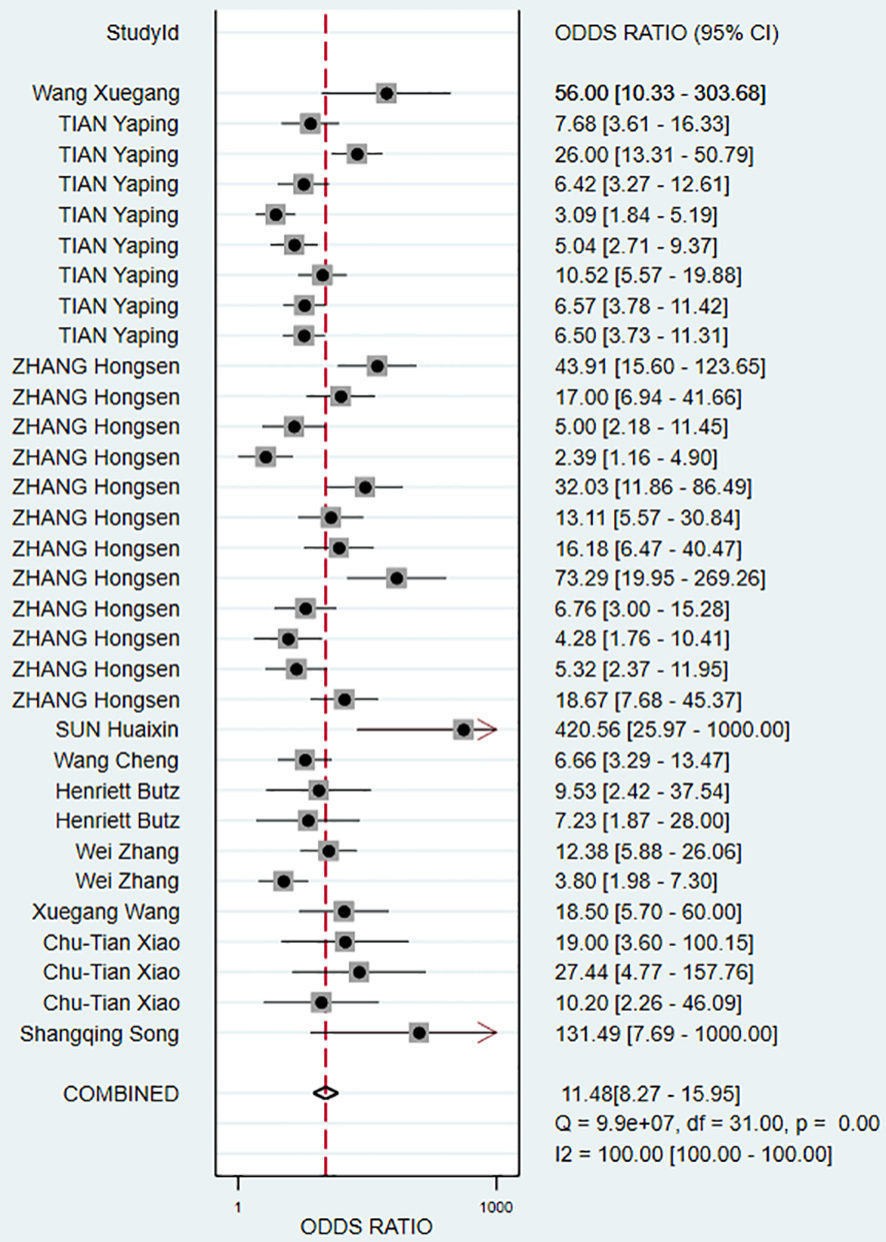
Forest plot demonstrating pooled DOR.

**Figure 6:**
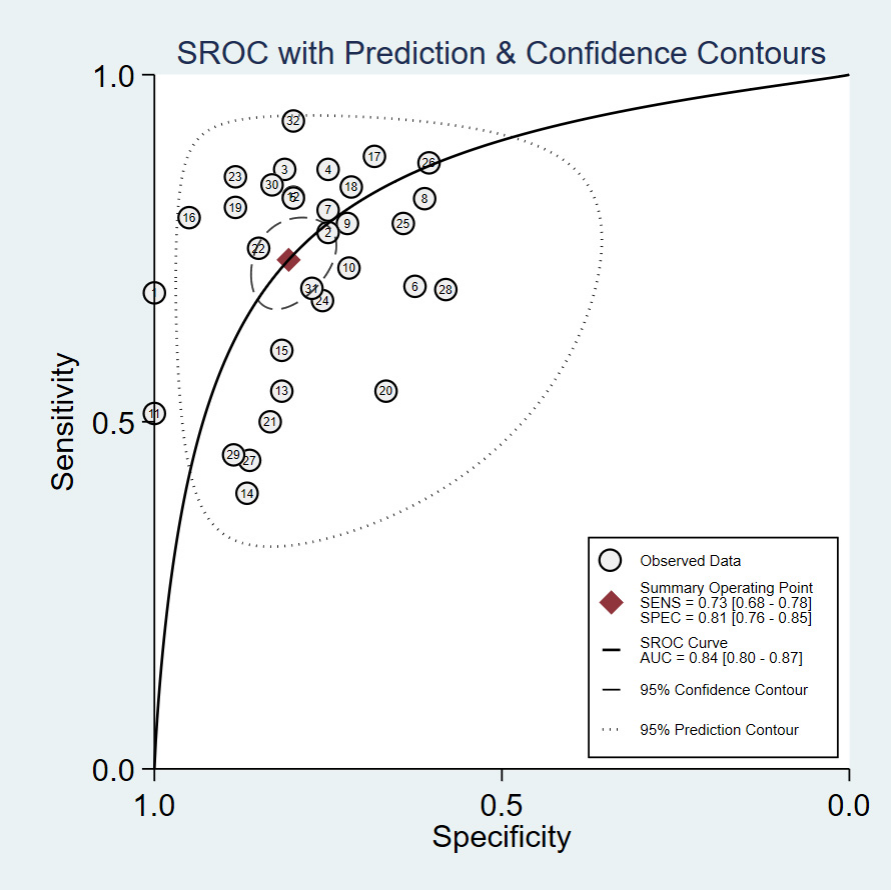
Receiver operating characteristic curve. The lables in the figure correspond respectively to **Table 1**.

The pooled Sen and Spe were 0.73 (95% *CI*, 0.68–0.78) and 0.81 (95% *CI*, 0.76–0.85), respectively (**Figure 3**). Compared with Sen and Spe, +LR and -LR are considered to have higher clinical value. The pooled +LR, -LR, and DOR were 3.80 (95% *CI*, 3.02–4.77), 0.33 (95% *CI*, 0.28–0.40), and 11.48 (95% *CI*, 8.27–15.95) respectively (**Figures 4**, **5**). The summary receiver operating characteristic curve (SROC) of the included studies showed an AUC of 0.84 (95% *CI*, 0.80–0.87), indicating good diagnostic accuracy (**Figure 6**).

### 3.4 Sensitivity analysis

**Figure 7:**
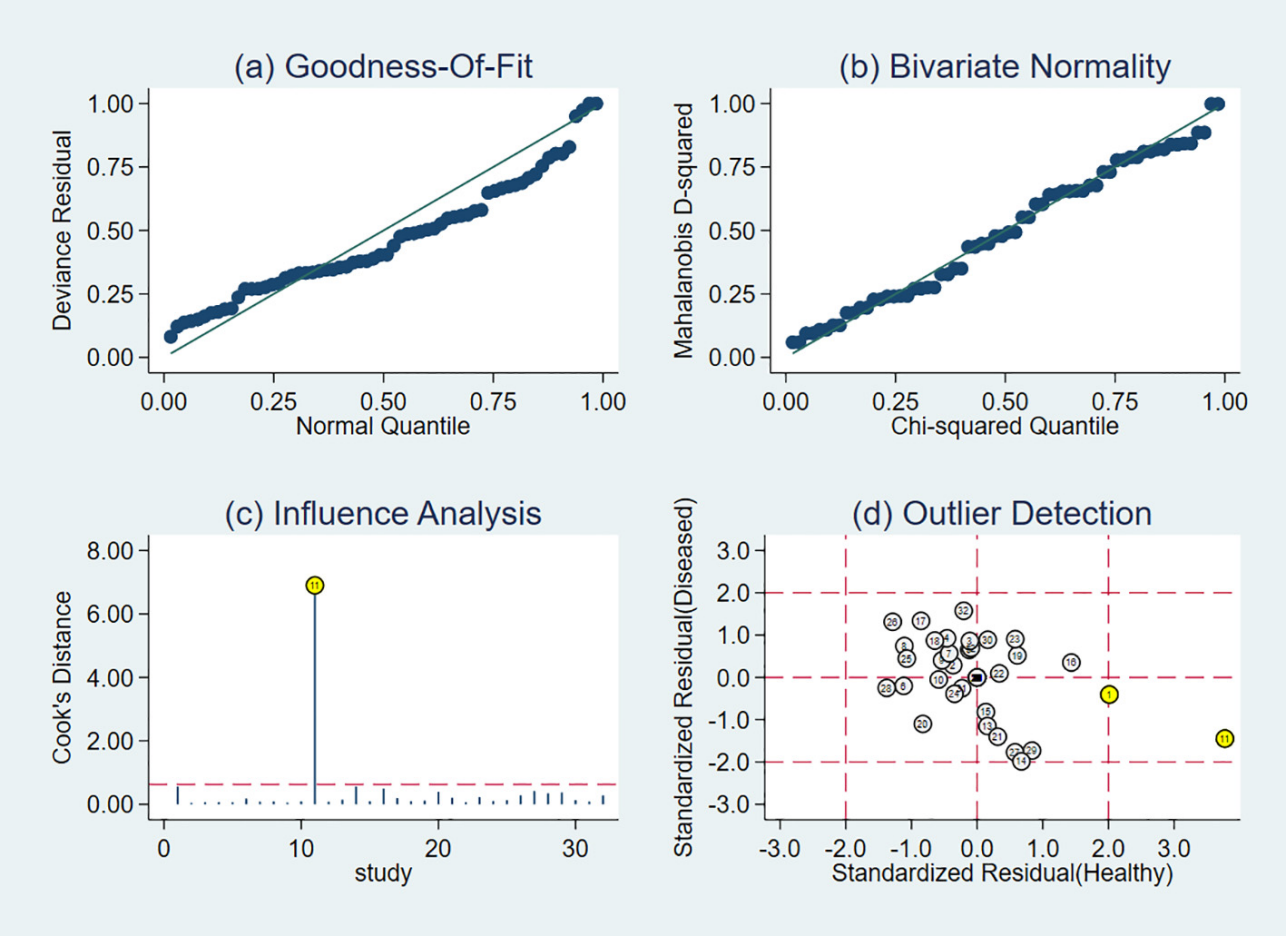
Results of sensitivity analysis. The lables in the figure correspond respectively to **Table 1**. **(A)** shows goodness of fit; **(B)** shows the bivariate normality test; **(C)** shows the impact analysis; **(D)** shows the outlier analysis.

Data from included studies were subjected to goodness-of-fit and binary normality analyses. The research was found to be reliable, and outlier detection revealed one anomalous value from a study by Shangqing Song (18). The study exhibited high sensitivity, and other primary studies do not lead to incorrect assessment of the computational results (**Figure 7**). After the aforementioned study was excluded, the pooled Sen increased by 1% to 0.74 (95% *CI*, 0.68–0.78), the pooled Spe increased by 3% to 0.80 (95% *CI*, 0.75–0.84), and the pooled DOR decreased by 0.51 to 10.97 (95% *CI*, 7.95–15.14). These changes suggested that the abovementioned study contributed to significant heterogeneity when compared with the other 10 studies.

### 3.5 Meta-regression and subgroup analyses

**Figure 8:**
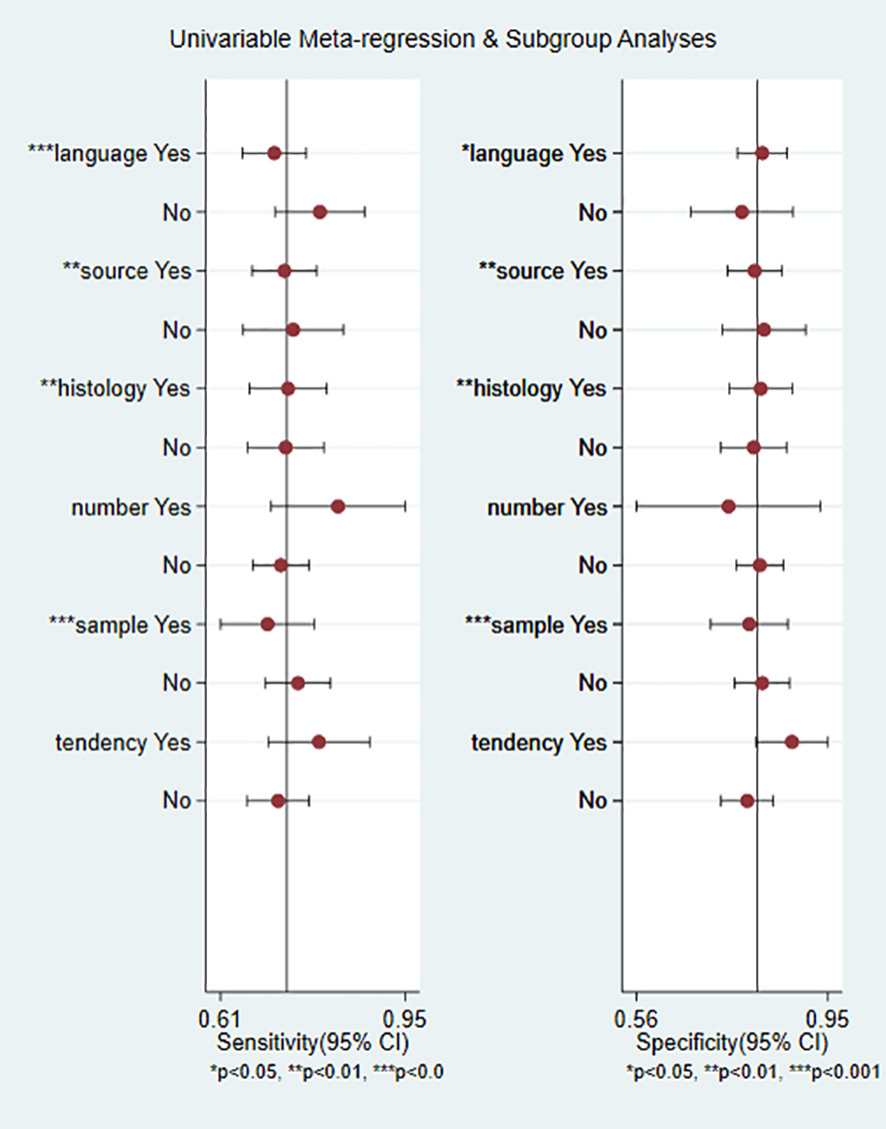
Results of meta-regression analysis. Language, publication language; source, source of exosomes, histology, histotype of RCC; number, the number of miRNA species carried by exosomes; sample, sample size of the included studies; tendency, miRNAs trend. * indicates a potential source of heterogeneity.

To determine the sources of heterogeneity caused by non-threshold effects, meta-regression analysis was performed using the publication language, source of exosomes, disease histotype, Exo-miRs quantity, miRNAs trend, and sample size as covariates. The results (**Figure 8**) showed that the publication language, source of exosomes, disease histotype, and sample size were potential sources of heterogeneity. On the contrary, the Exo-miRs quantity and miRNAs trend did not significantly contribute to heterogeneity.

Subgroup analysis was performed to verify heterogeneity among the included articles. Based on the source of exosomes, 3 studies with 166 cases were included in the urinary exosome group, whereas 9 studies with 810 cases were included in the blood exosome group(Zhang Hongsen2022 (25) use both urinary and blood exosomes). The Sen (0.74 vs. 0.73) and Spe (0.82 vs. 0.80) of both groups were similar. Based on the miRNAs trend, 8 studies with 517 cases were in the upregulation group, whereas 5 studies with 481 cases were in the downregulation group (Chu Tianxiao2020 (19), Zhang Hongsen2022 (25) included both upregulation and downregulation group). Sen (0.79 vs. 0.72) and Spe (0.91 vs. 0.78) were higher in the downregulation group than in the upregulation group. Additionally, the DOR was significantly higher in the downregulation group (39.98 vs. 9.26). Based on the publication language, 6 studies with 661 cases were in the Chinese group, whereas 5 studies with 247 cases were in the English group. Studies published in English exhibited higher Sen (0.77 vs. 0.71) but lower Spe (0.77 vs. 0.82) than those published in Chinese. In addition, the heterogeneity of studies published in English was less than 50%(15.94%). Based on the Exo-miRs quantity, 10 studies included only 1 type of Exo-miRs, whereas 1 studies included multiple types of Exo-miRs (Henriett Butz2015 (22) and Tian Yaping2018 (26) included multiple types of Exo-miRs). The Sen of the two groups was 0.72 and 0.83 respectively, and the specificity was 0.81 and 0.79 respectively. The results showed a significant difference in Sen, with higher sensitivity being observed among studies including multiple types of Exo-miRs. Additionally, the heterogeneity of sensitivity among these studies was 0, and the AUC value was 0.91. However, owing to the limited number of included studies, the presence of heterogeneity could not be ruled out. Based on the sample size of included studies (with a cutoff of ≥150 cases), the results showed the following Sen and Spe: 0.75 vs. 0.70 and 0.81 vs. 0.80. Based on the histological subtype of renal cancer, 3 articles with 169 cases were included in the RCC group, whereas 8 articles with 739 cases were included in the ccRCC group. The sensitivity of the two groups was 0.74 and 0.73, respectively, whereas the specificity was 0.81 and 0.81 respectively (**Table 2**).

**Table 2:** Results of subgroup analysis.

indicator	source of exosomes		miRNA trend		publication language		quantity of Exo-miRs		sample		histology	
	urine	blood	upregulation	downregulation	Chinese	English	1	>1	<150(n)	≥150(n)	RCC	ccRCC
literature volume (n)	3	9	8	5	6	5	10	2	7	4	3	8
case count (n)	166	810	517	481	661	247	880	154	328	580	169	739
Sen(95%*CI*)	0.74(0.65-0.81)	0.73(0.66-0.79)	0.72(0.66-0.77)	0.79(0.69-0.87)	0.71(0.64-0.77)	0.77(0.72-0.82)	0.72(0.67-0.77)	0.83(0.77-0.88)	0.75(0.69-0.81)	0.70(0.60-0.78)	0.74(0.66-0.80)	0.73(0.66-0.79)
*I^2^* (%)	81.68	90.61	88.80	91.42	91.26	15.94	89.29	0	84.07	92.92	86.32	91.11
Spe (95%*CI*)	0.82(0.73-0.88)	0.80(0.74-0.85)	0.78(0.74-0.82)	0.91(0.67-0.98)	0.82(0.76-0.87)	0.77(0.67-0.85)	0.81(0.76-0.86)	0.79(0.72-0.85)	0.81(0.77-0.85)	0.80(0.66-0.89)	0.81(0.76-0.85)	0.81(0.70-0.88)
*I^2^* (%)	73.36	87.82	79.71	93.45	88.40	58.65	86.79	57.7	59.16	93.12	60.50	91.03
PLR(95%*CI*)	4.10(2.67-6.30)	3.67(2.82-4.78)	3.31(2.77-3.96)	9.03(2.18-37.34)	3.91(2.96-5.15)	3.35(2.28-4.93)	3.87(3.01-4.98)	3.23(1.81-5.79)	4.04(3.26-5.01)	3.47(2.10-5.74)	3.88(3.06-4.92)	3.79(2.47-5.82)
NLR(95%*CI*)	0.32(0.23-0.44)	0.34(0.27-0.42)	0.36(0.29-0.44)	0.23(0.16-0.32)	0.35(0.28-0.44)	0.29(0.23-0.37)	0.34(0.28-0.41)	0.22(0.16-0.31)	0.30(0.24-0.39)	0.38(0.30-0.48)	0.33(0.25-0.43)	0.33(0.27-0.42)
DOR(95%*CI*)	12.98(6.67-25.29)	10.87(7.51-15.73)	9.26(6.69-12.71)	39.98(11.27-141.83)	11.10(7.48-16.50)	11.37(6.58-19.65)	11.37(7.97-16.21)	14.54(6.15-34.40)	13.38(9.04-19.81)	9.17(5.12-16.41)	11.93(7.57-18.79)	11.33(6.77-18.98)
AUC	0.85	0.83	0.82	0.88	0.83	0.82	0.83	0.91	0.86	0.80	0.85	0.82

Sen, Sensitivity; Spe, Specificity.

### 3.6 Publication bias

**Figure 9:**
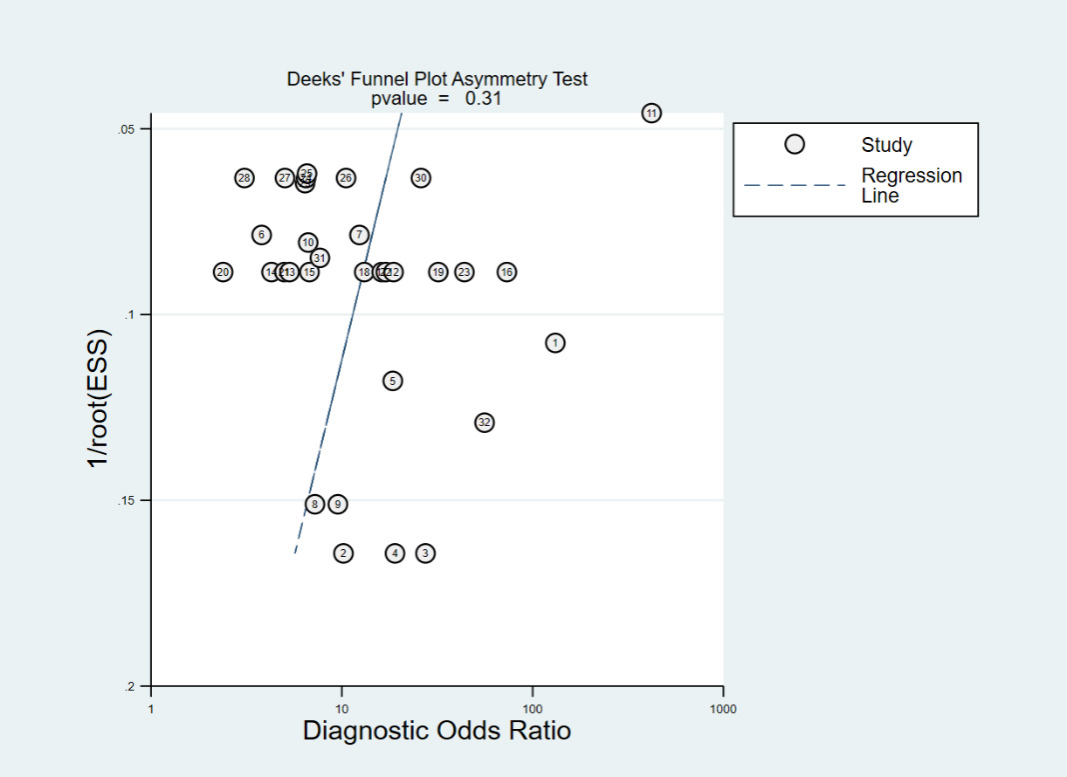
Deeks’ funnel plot for assessing publication bias. The lables in the figure correspond respectively to **Table 1**.

The Deeks’ funnel plot is commonly used to assess publication bias. In this meta-analysis, the funnel plot exhibited symmetry, with a *p*-value of 0.31 (>0.05), indicating no evidence of publication bias among the included studies. The results are shown in **Figure 9**.

### 3.7 Evaluation of clinical utility

**Figure 10:**
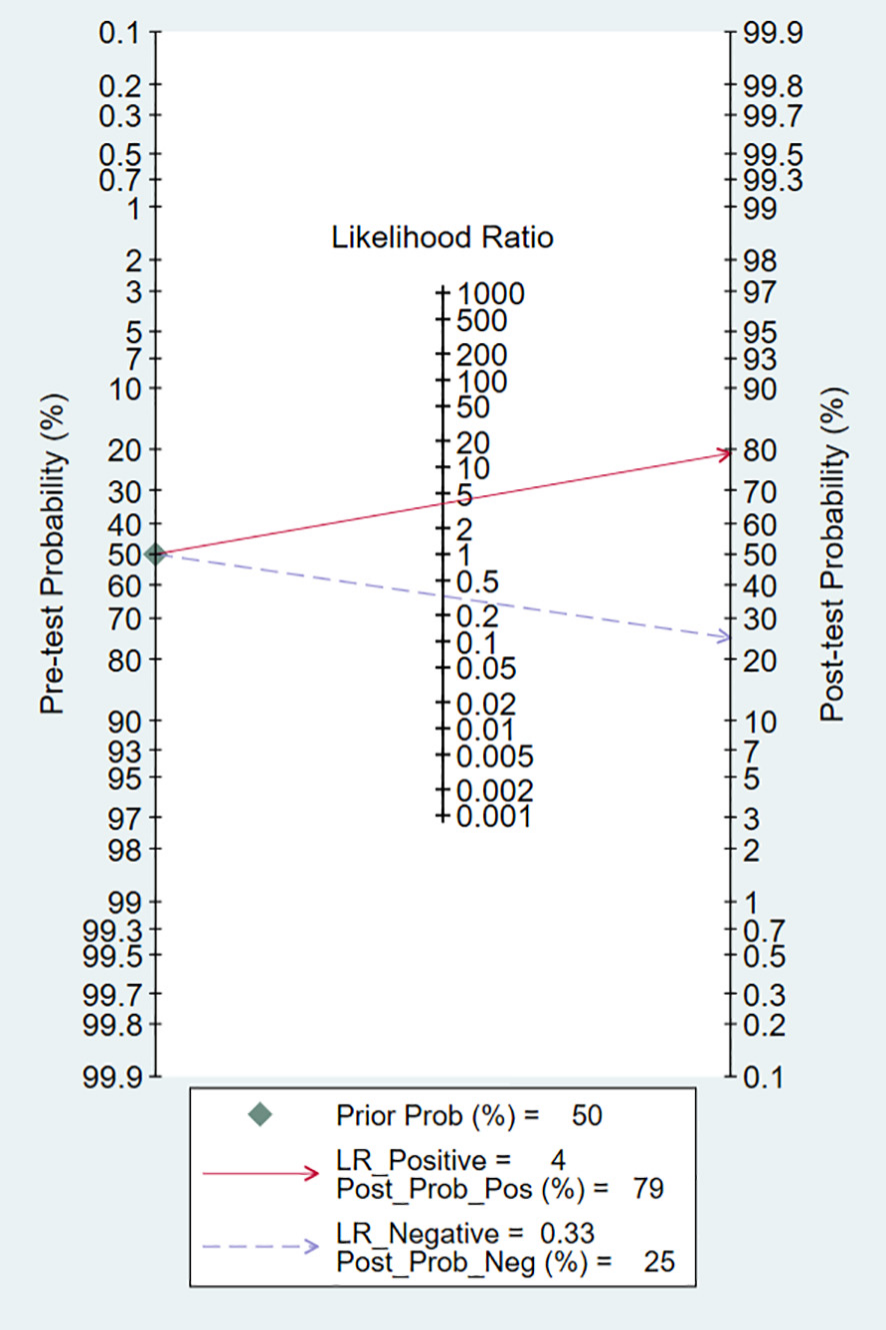
Fagan’s nomogram for assessing the diagnostic value of exosomal microRNAs.

Technological developments primarily rely on clinical problems and the clinical utility of the technology. In this meta-analysis, the pretest probability of DLR and PLR was set at 50% to assess the clinical value of Exo-miRs in the diagnosis of RCC (**Figure 10**). A positive result increased the post-test probability of having cancer to 79%, whereas a negative result decreased the post-test probability to 25%. These results indicate that Exo-miRs can be used as non-invasive biomarkers to complement existing diagnostic methods.

## 4 Discussion

### 4.1 Synthesis of evidence

The incidence of RCC has increased in recent years, with cases being reported across different age groups. RCC is often referred to as a “silent tumor” without evident symptoms in the early stages. Most patients with RCC have advanced disease at diagnosis. Only approximately 6%–7% of patients exhibit the classic triad of symptoms (abdominal pain, abdominal mass, and gross hematuria). Owing to substantial differences in treatment approaches and prognosis among RCC subtypes, early detection is crucial. Although surgical methods for RCC have been continuously updated in recent years, which can effectively improve kidney function and enhance patients’ survival outcomes (29), the mortality rate would significantly decrease if tumors could be detected and identified before the spread of cancer cells. The accuracy of ultrasonography and CECT in early diagnosis is limited owing to issues such as low sensitivity and adverse reactions. Therefore, the early detection rate of RCC is low, and the time to diagnosis is prolonged. The use of biomarkers for molecular-level screening may help achieve early diagnosis and design individualized treatment strategies for RCC patients. In addition, specific biomarkers may assist in the characterization of RCC.

miRNAs are involved in various physiological and pathological processes. It can be stably expressed in body fluids, such as blood and urine, with an average half-life of 119 hours (30, 31). Owing to the stability, Exo-miRs are considered as valuable biomarkers and have shown great application potential in early diagnosis, disease monitoring, prognosis evaluation, and personalized medicine. In particular, they have demonstrated high diagnostic value in various diseases, including glioma (32), gastric cancer (33), lung cancer (34), breast cancer (35), and testicular cancer (36). In addition, they have been identified as novel biomarkers for various urinary tract diseases, such as bladder cancer and prostate cancer (37, 38). Free circulating miRNAs in the body are mostly released after cellular apoptosis or necrosis. Although they are highly stable in blood and urine, when exposed to these body fluids for a long time, they can be degraded by endogenous ribonucleases, at the same time they are lack sensitivity and specificity (39). Exosomes possess a lipid bilayer, which enables them to ensure the integrity and functionality of bioactive molecules during intercellular communication. Owing to these unique features, extracellular vesicles hold substantial promise in disease diagnosis. The lipid bilayer characteristics of exosomes enable them to ensure the integrity and function of active molecules when they transmit information. Compared with free miRNA detection, a large number of fresh samples are required, and exosomes can be stored at -20°C for 5 years without destruction of content components (40). These characteristics make them unique advantages in disease diagnosis.

At present, the detection of exosomes mainly includes electron microscopy, flow cytometry, nanoparticle tracking and other technologies (41). Among them, nanoparticle tracking technology has higher sensitivity and specificity, which can achieve rapid and accurate detection of exosomes (42). The quantitative assessment of Exo-miRs is crucial for disease diagnosis and longitudinal monitoring. The detection technologies for Exo-miRs and protein biomarkers are continually advancing. Through bioinformatics analyses, including proteomics and RNA sequencing, the protein and miRNA profiles of exosomes are thoroughly examined. Real-time quantitative PCR has emerged as a widely adopted method for assessing miRNA and mRNA expression levels (43–45). In addition, there are some emerging detection methods, such as using molecular beacon (46), and CRISPR/Cas13a sensing system to detect Exo-miRs without amplification and extraction (47), which makes the detection of salivary GCF exosomes more advanced and sensitive.

Currently, extensive research is being conducted on biomarkers. In addition to Exo-miRs, MUC1 and other factors have emerged as significant areas. MUC1, a high molecular weight glycoprotein, has been identified as a potential biomarker for RCC, which including cellular proliferation, metabolic reprogramming, and angiogenesis—processes that are essential for the progression of RCC (48). Furthermore, research has demonstrated that the expression of MUC1 on exosomes may influence immune cell proliferation within the tumor microenvironment (49). Both MUC1 and Exo-miRs are related to the occurrence and development of RCC, and are of great value in the diagnosis and treatment. Blood-based biomarkers such as carcinoembryonic antigen and M2 pyruvate kinase have been evaluated for their potential in early detection of RCC. However, the low specificity and sensitivity limit their clinical utility.

Currently, liquid biopsy primarily focuses on molecular markers in blood to identify circulating tumor cells (CTCs), circulating tumor DNA (ctDNA), and exosomes. CTCs can be utilized for efficacy assessment and postoperative monitoring. However, a significant limitation is the insufficient quantity of CTCs in blood, which hampers effective early routine detection. ctDNA offers comprehensive insights into tumor progression but faces limitations due to its susceptibility to degradation in the bloodstream (50). Traditional RCC detection biomarkers include PAX8, CAIX, AE1/AE3, etc. (51). They usually need to be obtained through tissue biopsy, which is somewhat invasive. As a liquid biopsy procedure, Exo-miRs offer a minimally invasive alternative. In addition, Exo-miRs can provide more dynamic information than traditional biomarkers on reflect biological changes in tumors.

This meta-analysis included 23 Exo-miRs. These Exo-miRs play an important role in the pathological progression of RCC. For example, overexpression of miR-30c-5p can inhibit tumor growth in nude mice and suppress the growth of ACHN cells (17), indicating its tumor-suppressor role in ccRCC. miR-21 is a commonly overexpressed oncogene in various cancers. Plasma-derived exosomal miR-21 can exert anti-inflammatory and anti-apoptotic effects by targeting the PDCD4/NF-κB and PTEN/AKT pathways in renal tubular epithelial cells (52). On the contrary, miR-210 can activate HIF-1 by targeting the SDHD gene. Upregulation of HIF-1 can promote changes in the expression of VEGF and miR-210, thereby influencing angiogenesis in kidney tumors (53). These findings suggest that Exo-miRs play dual roles in RCC, as they can both promote and inhibit the growth of tumor cells. Therefore, research on Exo-miRs is important to develop strategies for early diagnosis and prompt treatment of RCC. It can also expand the applications of Exo-miRs in the fields of tissue engineering and regenerative medicine for other kidney diseases. With the continuous progress of research into the intrinsic mechanisms of Exo-miRs, the findings may revolutionize the diagnosis and treatment of genitourinary tumors. In particular, Exo-miRs may not only serve as non-invasive biomarkers for early diagnosis of RCC but also be targeted to modulate their expression to yield the desired therapeutic effects.

This meta-analysis included 11 studies with 1,646 samples, including 908 cases and 738 controls. The results indicated that Exo-miRs served as biomarkers for the diagnosis of RCC with high sensitivity and specificity. The probability of detecting abnormal Exo-miRs in patients with RCC was 3.8 times higher than that in healthy individuals, with the false-positive rate for 33%. Subgroup and regression analyses showed that studies published in English had higher sensitivity than those published in Chinese. Studies involving the use of exosomes carrying multiple miRNAs had higher sensitivity than those involving the use of exosomes with a single miRNA. In addition, studies including downregulated miRNAs had higher sensitivity than those upregulated miRNAs. Heterogeneity among the included studies was mainly attributed to the publication language, source of exosomes, disease histotype, and sample size of included studies. The number of included studies was limited, especially for subgroup analysis. Moreover, the predominance of single-center studies might have increased heterogeneity.

Although research on Exo-miRs diagnosis of RCC is still in its infancy and has not yet been widely applied in clinical practice, with continuous research and technological advancements, Exo-miRs are expected to become an important pathway. Building on further exploration of the mechanisms of exosome action and optimizing treatment plans to improve therapeutic effects, exosomes are expected to become an important pathway for the diagnosis of RCC, bringing more precise and effective treatment options to patients. Of course, optimizing the path of exosomes in medical applications requires addressing several core challenges. First, large-scale production must be realized to counteract the current low efficiency exosome production, which is fundamental to the widespread application of exosome-based therapeutic strategies. Second, it is essential to ensure the collection of high-quality and consistent exosomes. The physicochemical properties and purity of exosomes are directly influenced by the separation techniques employed, and so far there is no recognized “gold standard” for the isolation of exosomes. Which make the exploration and optimization of these methods to obtain stable quality a crucial step in advancing their clinical utility. Third, standardizing storage conditions is equally important. Maintaining the activity and stability of exosomes during storage is vital for the success of subsequent therapeutic applications. Lastly, further development of the therapeutic potential of exosomes is the ultimate goal. To address the limited efficacy of exosomes themselves, strategies such as overexpression or enrichment of therapeutic biomolecules can effectively enhance their therapeutic effects, paving new avenues for the broad application of exosomes in the medical field.

The application of Exo-miRs as diagnostic tools in clinical practice is still faces the impact of costs. As an emerging diagnostic tool, the development process of Exo-miRs requires a substantial financial investment, including various stages such as basic research, clinical trials, and technological optimization. The steps of Exo-miRs extraction, isolation, purification, and quantitative analysis require specific techniques and equipment, which may lead to higher production costs. In addition, the stability of Exo-miRs and storage conditions (such as the need for storage at -80°C) may also increase the costs of storage and transportation. Despite the aforementioned costs, Exo-miRs as diagnostic tools have higher sensitivity and specificity. Compared with existing diagnostic methods, they are non-invasive and convenient, capable of early diagnosis and providing more accurate diagnostic results. This can reduce repeated testing and unnecessary treatments. Overall, although the integration of exosomes as diagnostic tools may bring higher costs in the initial stage, in the long run, they can improve diagnostic accuracy and reduce unnecessary medical expenses, thereby reducing overall medical costs in the long term.

### 4.2 Limitations

(1) The research on using Exo-miRs for the diagnosis of RCC is relatively limited, often lacking relevant data. Consequently, the small number of included articles in this meta-analysis might have resulted in partial heterogeneity. Additionally, given the limited sample size, this may restrict the generalizability of the findings. Differences in the sampling and testing methods as well as limited geographical diversity, may lead to significant heterogeneity. (2) The QUADAS-2 tool was used to assess the methodological quality of all articles. The results showed that the included articles were of moderate-to-high quality, supporting the findings. However, during assessment, certain limitations in the design and execution were found. These limitations may impede the unbiased interpretation of the findings. First, it is noteworthy that a majority of the studies included healthy individuals as the control group. This approach, although common, may not accurately reflect the complexity of a cancerous state and may lead to an overestimation the diagnostic potential. To address this limitation, future studies should use tumor or benign tumor models and investigate the diagnostic efficacy of Exo-miRs in early-stage disease. (3) Owing to the limited number of included articles, the correlation between Exo-miRs and pathological stages or specific histological subtypes could not be analyzed. Therefore, the quality of the included article needs to be improved. In the future research, we will focus on collecting larger scale, multi-center and multi-regional sample data to enhance the wide applicability and statistical significance of the research conclusions. (4) The use of ROC curves determine the diagnostic accuracy of Exo-miRs was prevalent among included studies. This method often lacks a pre-specified threshold and validation in independent cohorts, which can decrease the robustness of the evidence presented. Therefore, future studies should incorporate validation techniques to evaluate the generalizability of statistical findings across different datasets. Additionally, to enhance internal and external validity, as well as the reproducibility of the results, future studies should strictly adhere to the updated guidelines established by the Standards for Reporting of Diagnostic Accuracy. Altogether, larger and prospective studies that focus on technical nuances and adhere to standardized protocols in reporting are warranted. Such studies are crucial for generating robust and reliable data that may guide clinical decision-making.

### 4.3 Practical significance and inspiration

Exo-miRs serve as potential biomarkers for the early diagnosis of RCC. Detection of Exo-miRs offers a non-invasive approach to obtaining biological information from patients, thus facilitating early diagnosis and individualized treatment and eventually improving the prognosis and quality of life of patients with RCC. Further research on Exo-miRs may elucidate the pathogenesis and disease course of RCC. Changes in the expression patterns of Exo-miRs may help understand their regulatory mechanisms and provide a theoretical basis for further investigation. Despite certain limitations, this meta-analysis highlights that the quality of data and the standardization of methods should be considered when conducting relevant studies. Improving the design and methodology of studies and controlling and reducing heterogeneity may help evaluate the potential of Exo-miRs as diagnostic and prognostic biomarkers and therapeutic targets, providing a more reliable basis for their use in clinical practice.

## 5 Conclusion

Based on quantitative analysis of data extracted from included articles, this meta-analysis suggests that Exo-miRs possess great potential in the diagnosis of RCC. The combination of multiple Exo-miRs or the combination of Exo-miRs with traditional biomarkers may represent an effective method for improving the diagnosis of RCC. This method is not only highly sensitive and specific but also non-invasive and radiation-free. Furthermore, Exo-miRs can be used as potential indicators of clinicopathological characteristics of RCC. However, given the limitations of this study, further validation through large-scale, multicenter, prospective, high-quality evidence-based clinical research is still needed to explore the mechanisms of action of Exo-miRs in RCC, standardize the sources of exosomes, and establish testing technology standards.

## Data Availability

The datasets presented in this study can be found in online repositories. The names of the repository/repositories and accession number(s) can be found in the article/supplementary material.
